# Ossicle in Anterior Cruciate Ligament: A Rare Occurrence

**DOI:** 10.1155/2014/616715

**Published:** 2014-04-13

**Authors:** Ashish Devgan, Reetadyuti Mukhopadhyay, Amanpreet Singh, Paritosh Gogna, Rohit Singla, Narender Kumar Magu

**Affiliations:** Department of Orthopaedics, Pt. B.D. Sharma PGIMS, Rohatk, Haryana 124001, India

## Abstract

The occurrence of an intra-articular ossicle is not rare in the knee, with reports suggesting the existence of meniscal osscile. There are also reports describing the attachment of the posterolateral bundle of the anterior cruciate ligament (ACL) to an accessory ossicle. However, despite an extensive search of the English literature we did not find much written about an intrasubstance ossicle in the ACL. We present the case of a 13-year-old male with an intrasubstance ossicle in the anteromedial bundle of the ACL of his right knee.

## 1. Introduction

The anterior cruciate ligament (ACL) consisting of its two bundles, the anteromedial and the posterolateral, attaches to the lateral femoral condyle on its posteromedial aspect. Very few variations are seen in the anatomy of the ACL [1].

It is not uncommon to find cysts within the ACL, their existence being around 1.3% on MRI studies [2]. The occurrence of postreconstruction and posttraumatic calcifications within the substance of ACL is also not rare [3].

However, a detailed search of literature failed to reveal much about the incidence or pathogenesis of an intra-ACL ossicle.

We, thus, present a case of a young male with chronic knee pain, with inconclusive history and physical examination, who on arthroscopic examination showed an ossicle within the substance of the anteromedial bundle of ACL.

## 2. Case Report

A 13-year-old student came to the outpatient department of our tertiary care institute complaining of pain along with clicking in his right knee for about 5-6 months. The patient was apparently well and had not noticed any discomfort in the involved knee more than 6 months back. He had been consulting local practitioners before he came to us, without much relief. The patient had mild pain in his right knee exaggerated by sporting activity and walking for long distances. He also complained of mild discomfort while walking. The pain had been nonprogressive for the past 5-6 months, with no relief with physiotherapy as was advised by the practitioners he had been consulting. He had absolutely no history of trauma to his knee or any swelling preceding the onset of the pain and discomfort. There was no history suggestive of any joint laxity or locking. The patient gave no history of any fullness or heaviness in his knee.

The examination of the patient was inconclusive. There was no fullness or swelling in the joint. The local temperature was comparable to the general body temperature, and no tenderness could be elicited. There was no ligament laxity, and the range of motion was normal with only slight pain and restriction of terminal extension. McMurray and Lachman were negative. The pain was exaggerated by making the patient climb up and down the stairs; it, however, did not restrict his activities.

**Figure 1:**
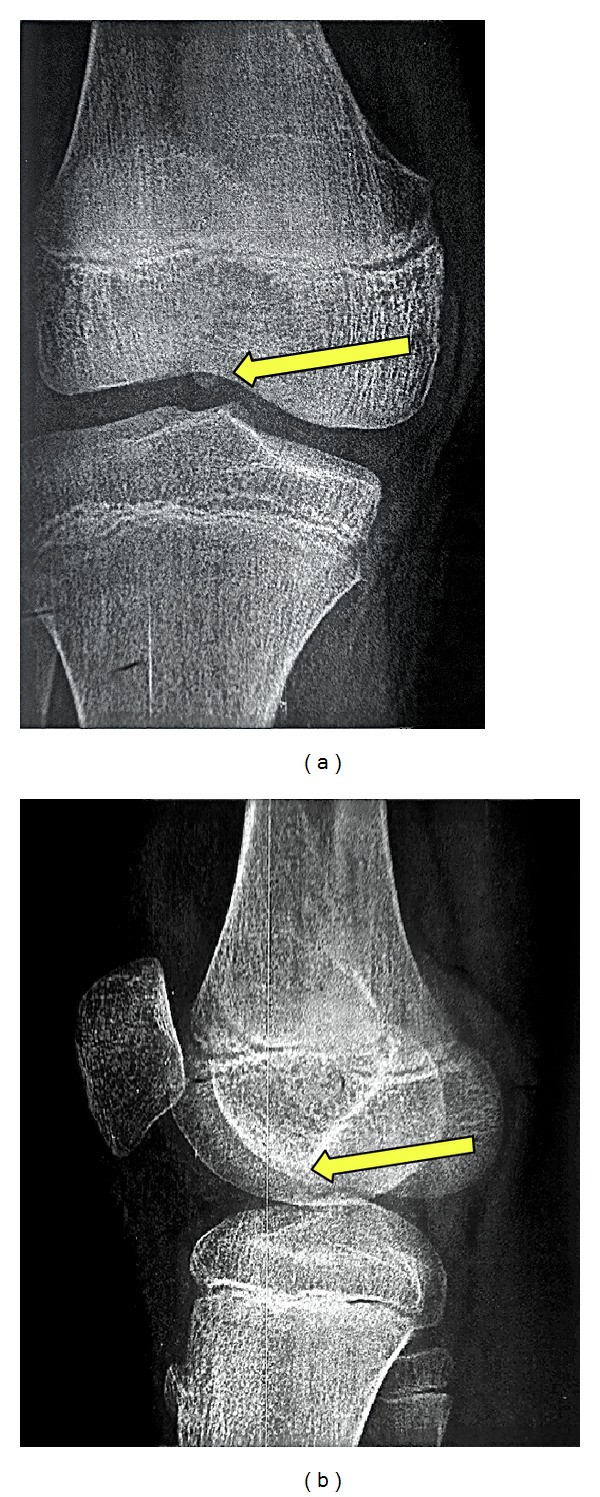
X-ray: (a) AP view and (b) lateral view showing what appeared to be a foreign body in the joint.

With an inconclusive history and physical examination the patient was advised one month of physiotherapy and limited sporting activity. He was asked to come back after a month for follow-up. A month later the patient came back with not much improvement in his symptoms. A plain X-ray although revealed something that appeared to be a loose body and was inconclusive (Figure 1).

**Figure 2:**
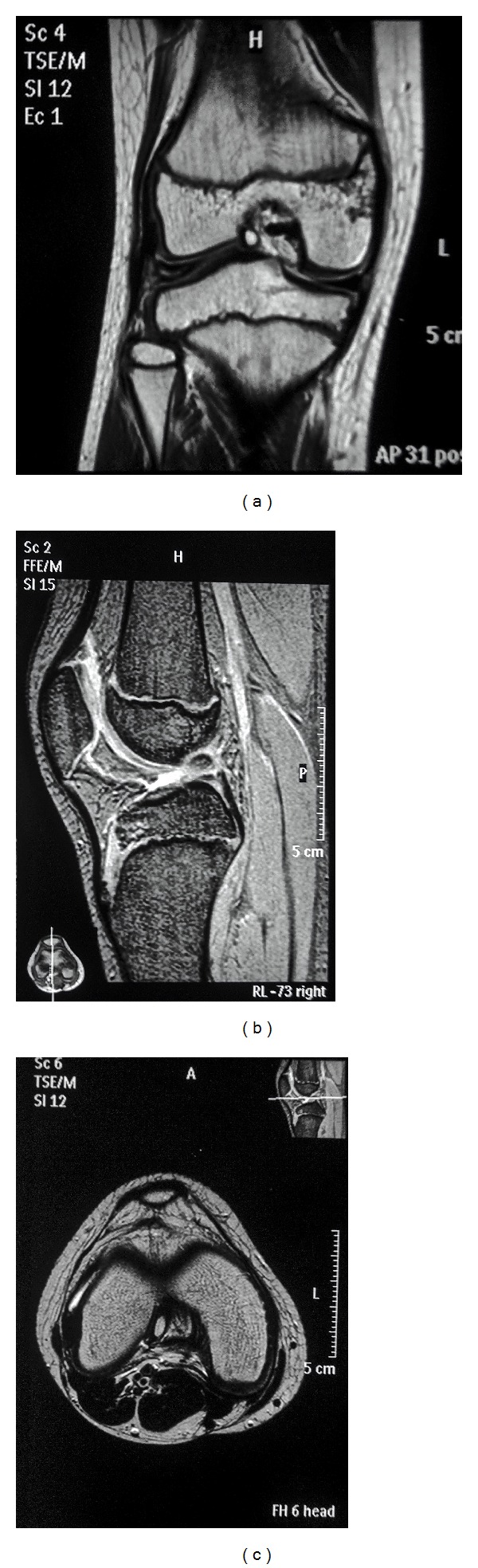
MRI: (a) coronal section, (b) sagittal section, and (c) axial section showing the ossicle within the substance of the anterior cruciate ligament.

An MRI was also advised and obtained. The MRI revealed a hypointense lesion of 1.3 cm by 0.8 cm, on both T_1_ and T_2_ images, in the substance of the anteromedial bundle of the ACL. The MRI also revealed a discoid lateral meniscus (Figure 2).

With a hypointense lesion in the MRI the diagnosis or nature of the lesion was yet in doubts. We thus decided to take a closer look into the lesion and the patient was worked up for a diagnostic arthroscopy and excision of the lesion.

**Figure 3:**
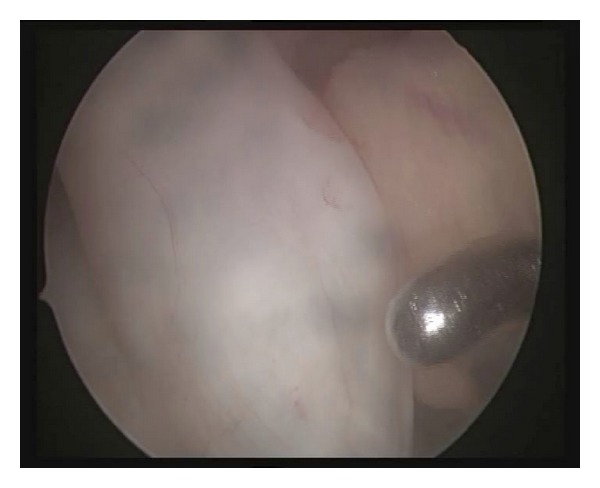
Arthroscopic view showing a swelling in the substance of the anteromedial bundle.

**Figure 4:**
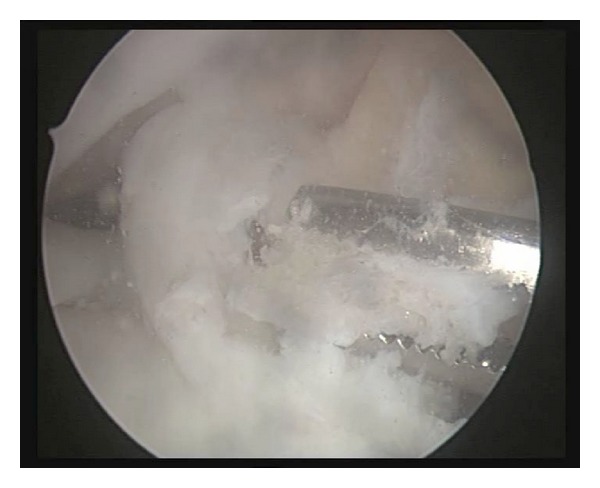
The intrasubstance ossicle being excised.

On entering the joint, a bulge in the substance of the anteromedial bundle of the ACL was evident, more clearly visible on 70° angle of the scope through the lateral portal (Figure 3). The rest of the joint appeared normal. It was swelling about 1.5 by 1 cm in size with the overlying tissue appearing normal. The rest of the ACL was also normal. The feel of the lesion was noncystic, rather hard. It was thus decided to slit open the ACL at the pathological site. On slitting it open an ivory white oval mass measuring about 1.5 cm by 1 cm was retrieved. The mass was excised and examined which appeared to be an ossicle within the ACL (Figure 4).

The patient is doing well after the excision of the ossicle. The patient is completely relieved of pain and has full range of movement in the involved knee. The terminal restriction of extension as was present on initial examination has also resolved. In a year of postoperative follow-up, the patient is asymptomatic and enjoying his sporting activities to the fullest.

## 3. Discussion

An ossicle may be defined as a mature lamellar and cancellous bone with a covering of hyaline cartilage and fatty marrow within [4]. Many authors have tried to describe an etiology for their occurrence. They may be vestigial organs or a result of ossification following mucoid degeneration [5, 6]. Traumatic etiology has also been put forward, suggesting their existence being that of a heterogeneous ossification [7]. There however exists a lot of confusion regarding their existence [5].

Following a detailed search of English literature, we could not come across any article talking about the occurrence of an ossicle within the substance of the ACL.

We however found mention of heterogeneous calcification in ACL following trauma or even ACL reconstruction [8].

Sarsilmaz and Gelal [1] had talked about a variation in the anatomy of the ACL, mentioning the attachment of the posterolateral bundle attaching to an accessory ossicle. The attachment to an intra-articular accessory ossicle, however, did not cause any knee instability.

The existence of intra-articular ossicles in knee is not very uncommon though. Meniscal ossicles although rare are not an infrequent occurrence [9]. Rohilla et al. [9] described a symptomatic meniscal ossicle in a 25-year-old male farmer.

Most patients with such intra-articular ossicles present with pain and on X-ray are most commonly misdiagnosed as loose bodies. However, the absence of any suggestive symptoms or signs made it unlikely that the mass could be a loose body [4].

MRI can be helpful in depicting the nature of the mass by localizing its site and showing isointensity to the normal bone marrow with a hypointense rim. It is a useful tool to differentiate it from other causes of loose bodies, chondrocalcinosis or osteochondritis dissecans. MRI also rules out the existence or absence of any other pathology in the joint [10].

The lack of its mention and rare occurrence in literature, however, held us back from making the diagnosis of an intra-ACL ossicle although the MRI findings were quite suggestive.

The occurrence of intra-ACL calcification leading to symptoms has been reported though. Tsujii et al [11] had described a symptomatic calcification of the ACL in a 31-year-old man with severe pain and movement restriction. The biopsy in their case, however, was suggestive of degenerative changes and resembled calcifying tendinitis.

Thus, based on the MRI appearance of the lesion and its physical appearance, we were prompted to make the diagnosis of an ACL ossicle.

## 4. Conclusion

The occurrence of intra-articular ossicles in the knee though have been reported, we did not come across any mention of an intrasubstance ossicle in the ACL. We thus report a case of an ossicle in the substance of the anteromedial bundle of the ACL, in a patient with mild pain and discomfort, who got completely relieved of his symptoms after its excision. After one year of postoperative follow-up, the patient is asymptomatic and back to his sporting activity without any constrains.
